# Supplementary material for: A Critical View of the Application of the APEX Software (Aqueous Photochemistry of Environmentally-Occurring Xenobiotics) to Predict Photoreaction Kinetics in Surface Freshwaters
Source: Molecules. 2019 Dec 18;25(1):9. doi: 10.3390/molecules25010009 (PMC7017383; doi:10.3390/molecules25010009)
Supplement: Supplementary file 1 [file molecules-25-00009-s001.zip › 3.2.4_gcc-4.4.0.zip!/3.2.4_gcc-4.4.0/3.2.4_gcc-4.4.0/doc/octave/html/interpreter/Nonlinear-Equations.html]

Nonlinear Equations - Untitled


Next: Diagonal and Permutation Matrices,
Previous: Linear Algebra,
Up: Top


---

## 19 Nonlinear Equations

Octave can solve sets of nonlinear equations of the form

```
     F (x) = 0
```

using the function `fsolve`, which is based on the Minpack
subroutine `hybrd`. This is an iterative technique so a starting
point will have to be provided. This also has the consequence that
convergence is not guaranteed even if a solution exists.

— Function File: **fsolve** (fcn, x0, options)  
— Function File: [x, fvec, info, output, fjac] **=** fsolve (fcn, ...)  
> Solve a system of nonlinear equations defined by the function fcn.
> fcn should accepts a vector (array) defining the unknown variables,
> and return a vector of left-hand sides of the equations. Right-hand sides
> are defined to be zeros.
> In other words, this function attempts to determine a vector x such
> that fcn `(`x`)` gives (approximately) all zeros.
> x0 determines a starting guess. The shape of x0 is preserved
> in all calls to fcn, but otherwise it is treated as a column vector.
> options is a structure specifying additional options.
> Currently, `fsolve` recognizes these options:
> `"FunValCheck"`, `"OutputFcn"`, `"TolX"`,
> `"TolFun"`, `"MaxIter"`, `"MaxFunEvals"`,
> `"Jacobian"`, `"Updating"` and `"ComplexEqn"`.
>
> If `"Jacobian"` is `"on"`, it specifies that fcn,
> called with 2 output arguments, also returns the Jacobian matrix
> of right-hand sides at the requested point. `"TolX"` specifies
> the termination tolerance in the unknown variables, while
> `"TolFun"` is a tolerance for equations. Default is `1e-7`
> for both `"TolX"` and `"TolFun"`.
> If `"Updating"` is "on", the function will attempt to use Broyden
> updates to update the Jacobian, in order to reduce the amount of jacobian
> calculations.
> If your user function always calculates the Jacobian (regardless of number
> of output arguments), this option provides no advantage and should be set to
> false.
>
> `"ComplexEqn"` is `"on"`, `fsolve` will attempt to solve
> complex equations in complex variables, assuming that the equations possess a
> complex derivative (i.e., are holomorphic). If this is not what you want,
> should unpack the real and imaginary parts of the system to get a real
> system.
>
> For description of the other options, see `optimset`.
>
> On return, fval contains the value of the function fcn
> evaluated at x, and info may be one of the following values:
>
> 1: Converged to a solution point. Relative residual error is less than specified by TolFun. 2: Last relative step size was less that TolX. 3: Last relative decrease in residual was less than TolF. 0: Iteration limit exceeded. -3: The trust region radius became excessively small.
>
> Note: If you only have a single nonlinear equation of one variable, using
> `fzero` is usually a much better idea.
>
>
>
> **See also:** fzero, optimset.
>
> Note about user-supplied jacobians:
> As an inherent property of the algorithm, jacobian is always requested for a
> solution vector whose residual vector is already known, and it is the last
> accepted successful step. Often this will be one of the last two calls, but
> not always. If the savings by reusing intermediate results from residual
> calculation in jacobian calculation are significant, the best strategy is to
> employ OutputFcn: After a vector is evaluated for residuals, if OutputFcn is
> called with that vector, then the intermediate results should be saved for
> future jacobian evaluation, and should be kept until a jacobian evaluation
> is requested or until outputfcn is called with a different vector, in which
> case they should be dropped in favor of this most recent vector. A short
> example how this can be achieved follows:
>
> ```
>           function [fvec, fjac] = user_func (x, optimvalues, state)
>           persistent sav = [], sav0 = [];
>           if (nargin == 1)
>             ## evaluation call
>             if (nargout == 1)
>               sav0.x = x; # mark saved vector
>               ## calculate fvec, save results to sav0.
>             elseif (nargout == 2)
>               ## calculate fjac using sav.
>             endif
>           else
>             ## outputfcn call.
>             if (all (x == sav0.x))
>               sav = sav0;
>             endif
>             ## maybe output iteration status, etc.
>           endif
>           endfunction
>           
>            ....
>           
>           fsolve (@user_func, x0, optimset ("OutputFcn", @user_func, ...))
> ```

Here is a complete example. To solve the set of equations

```
     -2x^2 + 3xy   + 4 sin(y) = 6
      3x^2 - 2xy^2 + 3 cos(x) = -4
```

you first need to write a function to compute the value of the given
function. For example:

```
     function y = f (x)
       y(1) = -2*x(1)^2 + 3*x(1)*x(2)   + 4*sin(x(2)) - 6;
       y(2) =  3*x(1)^2 - 2*x(1)*x(2)^2 + 3*cos(x(1)) + 4;
     endfunction
```

Then, call `fsolve` with a specified initial condition to find the
roots of the system of equations. For example, given the function
`f` defined above,

```
     [x, fval, info] = fsolve (@f, [1; 2])
```

results in the solution

```
     x =
     
       0.57983
       2.54621
     
     fval =
     
       -5.7184e-10
        5.5460e-10
     
     info = 1
```

A value of `info = 1` indicates that the solution has converged.

The function `perror` may be used to print English messages
corresponding to the numeric error codes. For example,

```
     perror ("fsolve", 1)
          -| solution converged to requested tolerance
```

When no Jacobian is supplied (as in the example above) it is approximated
numerically. This requires more function evaluations, and hence is
less efficient. In the example above we could compute the Jacobian
analytically as

```
     function J = jacobian(x)
       J(1,1) =  3*x(2) - 4*x(1);
       J(1,2) =  4*cos(x(2)) + 3*x(1);
       J(2,1) = -2*x(2)^2 - 3*sin(x(1)) + 6*x(1);
       J(2,2) = -4*x(1)*x(2);
     endfunction
```

The Jacobian can then be used with the following call to `fsolve`:

```
     [x, fval, info] = fsolve ({@f, @jacobian}, [1; 2]);
```

which gives the same solution as before.

— Function File: [x, fval, info, output] = **fzero** (fun, x0, options)  
> Find a zero point of a univariate function. fun should be a function
> handle or name. x0 specifies a starting point. options is a
> structure specifying additional options. Currently, `fzero`
> recognizes these options: `"FunValCheck"`, `"OutputFcn"`,
> `"TolX"`, `"MaxIter"`, `"MaxFunEvals"`.
> For description of these options, see optimset.
>
> On exit, the function returns x, the approximate zero point
> and fval, the function value thereof.
> info is an exit flag that can have these values:
>
> - 1
>   The algorithm converged to a solution.- 0
>     Maximum number of iterations or function evaluations has been exhausted.- -1
>       The algorithm has been terminated from user output function.- -2
>         A general unexpected error.- -3
>           A non-real value encountered.- -4
>             A NaN value encountered.
>
>
>
>
> **See also:** optimset, fsolve.
